# Audio-Visual Speech Cue Combination

**DOI:** 10.1371/journal.pone.0010217

**Published:** 2010-04-16

**Authors:** Derek H. Arnold, Morgan Tear, Ryan Schindel, Warrick Roseboom

**Affiliations:** School of Psychology, The University of Queensland, St. Lucia, Queensland, Australia; University of New South Wales, Australia

## Abstract

**Background:**

Different sources of sensory information can interact, often shaping what we think we have seen or heard. This can enhance the precision of perceptual decisions relative to those made on the basis of a single source of information. From a computational perspective, there are multiple reasons why this might happen, and each predicts a different degree of enhanced precision. Relatively slight improvements can arise when perceptual decisions are made on the basis of multiple independent sensory estimates, as opposed to just one. These improvements can arise as a consequence of probability summation. Greater improvements can occur if two initially independent estimates are summated to form a single integrated code, especially if the summation is weighted in accordance with the variance associated with each independent estimate. This form of combination is often described as a Bayesian maximum likelihood estimate. Still greater improvements are possible if the two sources of information are encoded via a common physiological process.

**Principal Findings:**

Here we show that the provision of simultaneous audio and visual speech cues can result in substantial sensitivity improvements, relative to single sensory modality based decisions. The magnitude of the improvements is greater than can be predicted on the basis of either a Bayesian maximum likelihood estimate or a probability summation.

**Conclusion:**

Our data suggest that primary estimates of speech content are determined by a physiological process that takes input from both visual and auditory processing, resulting in greater sensitivity than would be possible if initially independent audio and visual estimates were formed and then subsequently combined.

## Introduction

Researchers often refer to multi-sensory integration, but some evidence cited for this is inconclusive. Two types of observation are often taken as evidence, subjective reports concerning changed perceptual content [1]–[6] and changes in the precision of perceptual decisions [7]–[9]. Neither necessarily provides unambiguous evidence for integration, for reasons that we outline below.

Of the two types of evidence mentioned, subjective reports concerning changed perceptual content is weakest. Subjective reports could change because sensory integration has taken place. Alternatively, this could happen because the provision of additional information disposes the observer to report a particular outcome. The latter possibility could be described as a decision-level sensory interaction – it does not necessitate integration. Rather, the two sensory codes could remain independent, with either shaping the perceptual decision process.

Improvements in the precision of perceptual decisions can provide stronger evidence, but here too findings can be ambiguous, as the provision of multiple independent sources of information can lead to improved sensitivity in the absence of sensory integration [10]–[13]. However, the degree of improvement in the precision of sensory decisions, when two cues are available as opposed to just one, can be diagnostic of the underlying computational process.

Slight improvements can arise when making a decision on the basis of two independent sources of information, as opposed to just one. These improvements are known as probability summation [10]–[13]. Probability summation can occur when redundant inputs are encoded by independent sensory systems. The advantage accrues because each system has an independent probability of exceeding the requisite threshold for an accurate sensory decision [10], [12], [14]. Importantly, probability summation does not necessitate sensory integration. Rather, it depends on there being two independent sensory estimates. Thus, in order to attribute precision improvements to an integration process, it is necessary to show that the improvements are greater than those that can be predicted on the basis of probability summation. In the absence of such evidence, it might be appropriately conservative to describe such data as being indicative of multi-sensory interaction, rather than of integration.

Greater improvements can be predicted via sensory integration, but here too at least two distinct computational processes might be responsible. For instance, sensory integration could be achieved via a summation of two initially independent sensory estimates. When the summation is weighted in accordance to the variance associated with each of the initially independent sensory estimates, the combination process is often described as a Bayesian maximum likelihood estimate (MLE) or an optimal integration [7]–[8]. This weighting enhances the sensitivity of perceptual decisions made on the basis of the combined estimate.

Even greater improvements can be predicted if two sensory cues are encoded by a common physiological process. In these circumstances sensitivity for the combined signals can be predicted by simply adding sensitivities for the isolated signals [10], [15]–[16]. This process is called linear summation and it can occur, for instance, when detecting small proximate spots of light.

We can attempt to determine whether sensitivity improvements in audio-visual speech discrimination are related to probability summation, MLE or to linear summation by employing a Minkowsky metric [15], [17] to evaluate the degree of summation. This can be calculated as follows:
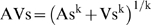



where AVs denotes sensitivity to combined audio and visual signals, As denotes sensitivity to audio signals, and Vs denotes sensitivity to visual signals. The exponent k signifies the degree of summation. A probability summation would correspond with k = 3–4. MLE improvements correspond with a quadratic summation, or k = 2. A linear summation, as observed when multiple signals are encoded by a single physiological mechanism [15]–[16], corresponds with k = 1. This last situation can only ensue when no independent sensory estimates are derived prior to the linear summation. The computational advantage of linear summation arises because the sensory estimate is subject to just one intrinsic source of neural noise, as opposed to at least two when independent sensory estimates are first determined.

To examine these issues we assessed audio-visual speech recognition. It is well established that audio-visual interactions can shape the apparent content of speech [3] and improve the sensitivity of speech recognition [9]. The question here is whether the AV improvement for speech recognition is consistent with probability summation, MLE or with a linear summation.

## Results

**Figure 1:**
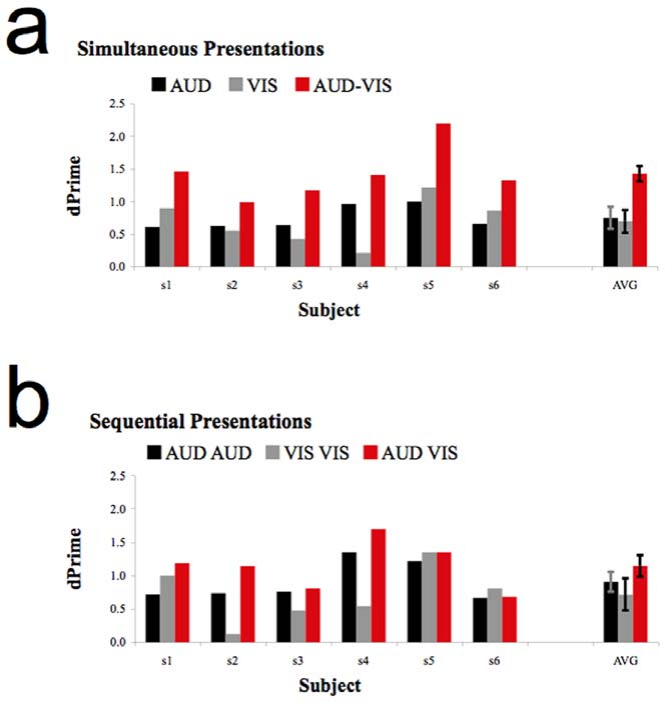
Bar plots showing d' sensitivities. (a) Bar plot showing sensitivities for AUD, VIS and AV presentations during Simultaneous runs of trials. Data are shown for each of six observers, along with the average performance across observers. Error bars depict +/− 1 SEM. Subjects 3, 4 & 5 are authors. Note that their data does not differ qualitatively from other participants (b) Data from Sequential runs of trials. Details are as above.

The experimental design involved either simultaneous or sequential presentations of concordant audio and visual (AV) speech cues. There were also single presentations of audio (AUD) and visual (VIS) speech cues, and sequentially repeated AUD AUD and VIS VIS presentations. Stimuli were customized for each participant, by adding requisite levels of noise, to ensure approximately equal sensitivity to single presentations of AUD and VIS speech cues (see Materials and Methods). Objective measures of speech recognition sensitivity, d' [18], were calculated for each participant (see Figure 1a-b). From single AUD and VIS presentations, we were able to calculate sensitivity *predictions*, assuming different levels of summation, for all other stimulus presentation conditions.

As can be seen in Figure 1a, there was a substantial sensitivity improvement for Synchronous AV speech relative to either single AUD (t5 = 5.42, p = 0.003) or VIS (t5 = 5.88, p = 0.002) presentations. Numerically, there was a smaller sensitivity improvement for Sequential AV speech. This reached significance in comparison to single AUD trials (t5 = 3.59, p = 0.016), but not in comparison to VIS trials (t5 = 1. 93, p = 0.111).

**Figure 2:**
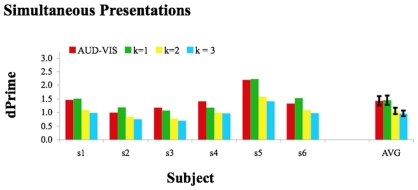
Bar plot depicting sensitivity during Simultaneous AUD-VIS trials (Red) and AV sensitivities predicted on the basis of different magnitudes of summation. Predictions are based on AUD and VIS sensitivities during Simultaneous trial runs (see Figure 1a). K = 1 corresponds with a linear integration prediction, k = 2 with a quadratic summation, and k = 3 with probability summation (see main text for details). Error bars depict +/− 1 SEM.

AV sensitivities during Simultaneous runs of trials (see Materials and Methods) are re-plotted in Figure 2. Along with the observed data, we have plotted predictions that assume different magnitudes of summation. These predictions are based on individual estimates of sensitivity for single AUD and VIS presentations (see Figure 1a). We found that Simultaneous AV sensitivity is well predicted via a linear summation (k = 1, t5 = .32, p = 0.759) but is inconsistent with either a probability summation (k = 3, t5 = 5.63, p = 0.002) or a MLE (k = 2, t5 = 6.25, p = 0.002).

**Figure 3:**
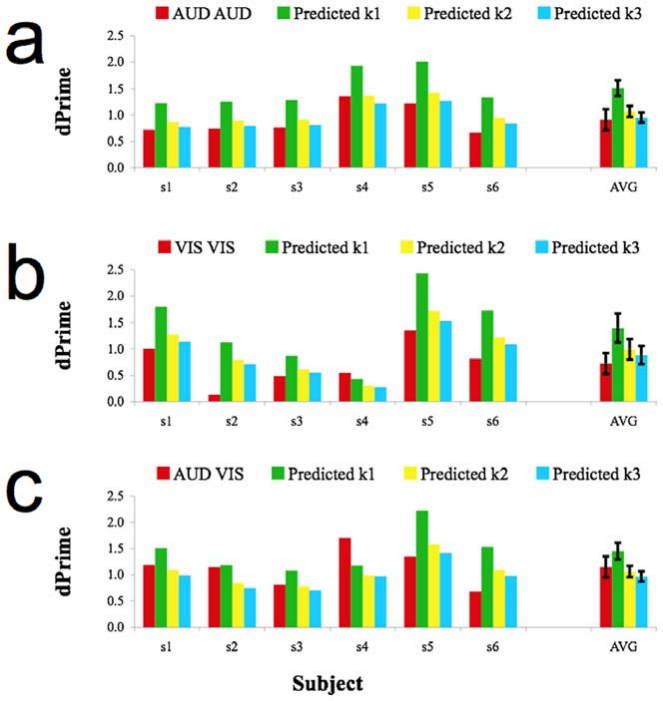
Bar plots depicting observed and predicted sensitivities. (a) Bar plot depicting sensitivity during Sequential AUD AUD trials (red) and AUD AUD sensitivities predicted on the basis of different magnitudes of summation. Predictions are based on AUD sensitivities during Simultaneous trial runs. (b) Bar plot depicting sensitivity during Sequential VIS VIS trials (red) and VIS VIS sensitivities predicted on the basis of different magnitudes of summation. Predictions are based on VIS sensitivities during Simultaneous trial runs. (c) Bar plot depicting sensitivity during Sequential AUD VIS trials (Red) and AUD VIS sensitivities predicted on the basis of different magnitudes of summation. Predictions are based on AUD and VIS sensitivities during Simultaneous trial runs. Error bars depict +/− 1 SEM.

We can also use individual estimates of sensitivity for single AUD and VIS presentations to predict performance in sequentially repeated presentations. Predictions made on this basis for sequential AUD AUD, VIS VIS and AUD VIS presentations are respectively depicted in Figure 3a–c. The trends in these data are informative. Both AUD AUD (k = 1, t5 = 12.75, p<0.001; k = 2, t5 = 4.42, p = 0.007; k = 3, t5 = 0.99, p = 0.37) and VIS VIS (k = 1, t5 = 3.61, p<0.015; k = 2, t5 = 2.15, p = 0.09; k = 3, t5 = 1.41, p = 0.29) are most consistent with k = 3, and therefore with probability summation. However, performance in sequential AUD VIS trials (k = 1, t5 = 1.42, p = 0.214; k = 2, t5 = 0.54, p = 0.615; k = 3, t5 = 1.23, p = 0.273) are most consistent with k = 2, and therefore with MLE.

## Discussion

Our data suggest that an integration process can result in heightened sensitivity for simultaneous AV speech cues. The magnitude of facilitation was well predicted by a linear summation, but could not be predicted on the basis of a probability summation [10], [11]–[12], or a Bayesian MLE [8], [19]. AUD and VIS signals in sequential trials were, however, less likely to become integrated as they were separated by a 3 second ISI (see Materials and Methods). Consistent with this, the facilitation of speech recognition for sequential AV signals was inconsistent with linear summation.

The maximum likelihood estimation (MLE) model is a popular contemporary approach to multi-sensory research [7]–[8], [19]. MLE assumes multi-sensory integration and has proven highly successful at predicting multi-modal sensitivity. As can be seen in Figures 2 and 3, it can be difficult to differentiate the predictions of MLE from those of probability summation [10]–[12]. Multimodal sensitivities that are consistent with one of these predictions will often be consistent with the other. As only one of these predictions assumes multi-sensory integration, this too is potentially problematic.

Our data concerning simultaneous AV speech sensitivity are consistent with a different form of integration that results in greater summated sensitivity. They are consistent with a linear summation of the two sources of information. They suggest the existence of a process for which AUD and VIS inputs are equitable. This is analogous to visual mechanisms that linearly summate different sources of information to form an integrated code [16].

The present results contrast with AV sensitivities in other contexts. Discriminations between different rates of AV flicker/flutter, for instance, are well predicted via MLE [11]. So too are data concerning AV spatial localization [7]. As can be seen in Figure 2a, our simultaneous AV speech data exceed MLE predictions. These data suggest that AUD and VIS speech cues are encoded by a common physiological mechanism, whereas independent processes determine initially independent spatial location [7] and change rate [11] estimates.

We speculate that the initial independence of sensory estimates for AUD and VIS locations might be functionally advantageous, allowing for attention to be directed at will toward auditory and or visual signal origins [20]. However, the determination of initially independent AUD and VIS location estimates necessarily limits the sensitivity of any subsequent code formed by summating these initial estimates. Such a code would be limited by the variance associated with each, initially independent, sensory estimation.

Greater sensitivity to multi-sensory input could be achieved if initial independent uni-modal sensory estimates are not computed. Instead, the initial sensory estimate could be determined via a process that takes direct input from multiple sensory modalities. This would maximize the precision of sensory decisions when multi-modal signals are in accord, but may come at a cost of being unable to ignore discordant multi-sensory input. We believe that previous research concerning AV facilitation of speech recognition [9], and an inability to ignore discordant visual cues when attending auditory speech [3], [21], are consistent with primary sensory estimates of speech content being based on both auditory and visual processing.

Some of the most compelling evidence for sensory integration is provided by cases wherein the integration process results in impaired sensitivity, due to the combination of conflicting sensory cues. In this context metamers can be generated [22]. These are composite stimuli that cannot be discriminated from physically differing inputs, even though their constituents could be. For instance, a red and or green light could easily be distinguished from yellow, but this can become impossible when the red and green lights are combined. The compelling aspect of this situation is the loss of sensitivity for the independent cues as a consequence of sensory integration.

Our data suggest that AUD-VIS speech might be an example wherein cross-modal integration results in a loss of sensitivity to constituent inputs. If primary estimates of speech content are derived via a process that takes direct input from audition and vision, a loss of sensitivity to the independent audio and visual speech cues would ensue because these cues are never encoded in isolation. Note, however, that this does not imply a misperception of mouth aperture, or of the tonal qualities of the voice. Rather, the misperception would be specific to speech content. The mandatory sensory fusion this suggests is certainly consistent with the striking perceptual distortions that ensue when discordant audio and visual cues are encountered [3], [21]. Thus a strong implication of our data is that it should be possible to create metamers for speech content.

While conflicting with many examples of AV sensitivity facilitation, our data are consistent with those reported for spatially and temporally co-localised AV directional movement [15]. This observation set a precedent for the linear summation of AV signals. Our results are also strikingly similar to a recent dataset concerning AV sensitivity to biological motion [23]. However, our speech recognition data contrast with AV movement sensitivity [15], in that our AUD and VIS speech signals did not have to be presented in strict spatial co-register to induce linear summation.

Sequential presentations were included in this study primarily as a control. One would not expect sensory level integration in these trials, as the sequential presentations were separated by three seconds and constituted clearly separate sensory events. It is therefore unsurprising that neither repeated unimodal nor sequential cross modal presentations resulted in performance consistent with linear summation. Instead, performance in these conditions was statistically consistent with probability summation, as expected when two independent sensory estimates serve as the basis for decision making [10]–[13]. However, there was an interesting trend, with many participants performing better in sequential AUD VIS trials than predicted by probability summation. Instead, performance in sequential AUD VIS trials was better predicted by MLE. We hasten to point out, however, that performance in sequential AUD VIS trials was statistically indistinguishable from *both* MLE and probability summation based predictions.

Our sequential AUD VIS data are reminiscent of an earlier data set, concerning discriminations between different rates of change [11]. In that study both simultaneous and sequential AUD VIS presentations resulted in enhanced sensitivity relative to unimodal presentations. Moreover, performances in both simultaneous and sequential cross modal presentations were consistent with MLE. When considered in conjunction with the trend in our data, this suggests that AUD VIS integration need not occur at a sensory level of processing. Instead, clearly differentiated sensory events are capable of promoting a level of performance consistent with integration via a weighted summation [7]–[8], [11]. This may imply that sensory estimates can become integrated via MLE at a decision-level of analysis.

The experimental conditions reported here were intentionally highly constrained. This allowed us to determine objective sensitivity measures, making it possible to differentiate the predictions of linear from other forms of summation. However, task success rested on the detection of a single phoneme difference. This is not representative of the unconstrained conditions, nor of the highly variable content, of naturalistic speech. It remains to be seen, therefore, if our approach can be adopted to account for the results of experiments that adopt less constrained conditions, so as to better approximate the conditions of naturalistic speech [24]–[25]. However, our data speak to the ability to discriminate between utterances based on the detection of single phoneme differences. Thus, while it remains to be seen how far our findings will generalize, our data are relevant for understanding speech comprehension.

The paradigm we have adopted could easily be developed to precisely estimate where the vital information for cross modal integration is located. For instance, a trial-by-trial record could be kept concerning the locations of the obscuring visual noise elements. Then, by correlating performance with noise element locations, the critical facial regions could be identified. By using dynamic noise elements, this investigation could be extended into the temporal domain. Similar manipulations could be applied to the auditory stimulus.

Our data reveal a dramatic improvement in speech recognition following exposure to coincident and concordant AUD and VIS speech cues, compared to performance with isolated AUD or VIS input. The magnitude of improvement is inconsistent with the formation of initially independent auditory and visual estimates of speech content. Instead, we suggest that primary estimates of speech content are determined by a process that takes direct input from visual and auditory processing.

## Materials and Methods

Six members of the University of Queensland Perception Lab participated in the experiment, 3 of the authors and an additional 3 who were naïve as to the purpose of the experiment. All reported normal, or corrected to normal, visual acuity and normal hearing. Before the experiment, participants were provided with an information sheet, which outlined the general purpose of the study and possible consequences of participation. Participants were also informed that they could withdraw at any time without penalty. The experiment only began once the participant had given verbal consent to the experimenter to continue. This experiment was approved by The University of Queensland School of Psychology ethics committee, and was conducted according to the principles of the Declaration of Helsinki.

Visual stimuli were displayed on a 19″ Sony Trinitron G420 monitor at a resolution of 1024×768 pixels, driven by a ViSaGe graphics card from Cambridge Research Systems at a refresh rate of 120 Hz. Observers viewed all stimuli from 57 cm with their head placed in a chinrest. Auditory stimuli were generated using Matlab software and were presented via Sennheiser HD 25-1 headphones at an intensity of ∼70 dbSPL.

Six movies of 3 of the authors uttering two phrases were recorded. The phrases were, ‘My name is Gary’ and ‘My name is Barry’. These were calibrated such that they were all of the same duration (2.03 secs) and the initiation of mouth movements took place at approximately the same movie epochs (see supplementary Movies S1, Movie S2, Movie S3, and Movie S4).

To equate unimodal audio and visual performance, audio and visual noises were added to the stimuli. Audio noise consisted of white noise, generated on a trial-by-trial basis. Visual noise was generated by partitioning the visual stimulus into 0.13dva square regions. A proportion of these were set to black (visual noise), whereas the animation could be viewed in others (visual signal). The positions of the black square regions were determined at random on a trial-by-trial basis.

Prior to the experimental procedures, estimates of the signal to noise ratio, at which observers were correct on 60% of uni-modal trials during forced choice (Was the actor's name Barry or Garry?) tasks, were determined. Audio S/N ratios referred to the amplitude of white noise in proportion to the peak amplitude of the auditory (AUD) signal. Visual (VIS) S/N referred to the proportion of un-obscured VIS animation. During a run of trials, both S/N ratios were manipulated according to the method of constant stimuli (VIS 0.10, 0.20, 0.25, 0.50, 0.75 or 1.00 AUD 0.08, 0.10, 0.13, 0.20, 0.40 or 1.00). During a run of trials, each S/N ratio was presented 12 times (6× Barry, 6× Gary). A run of trials therefore involved 72 AUD and 72 VIS trials, 144 individual trials in total, all completed in a random order. Each participant completed a single preliminary run of trials. Estimates were then determined by fitting Weibull functions to individual data.

In the subsequent experiment, runs of trials were grouped according to the style of stimulus presentation. During simultaneous runs of trials, AUD, VIS or AV signals were presented and observers were required to indicate if the speaker had said that their name was Barry or Gary, by pressing one of two CB6 (Cambridge Research Systems) response buttons.

AUD trials involved the presentation of the AUD signal accompanied by a static picture of the speaker (the first frame of the animation), partially obscured by VIS noise elements. VIS trials involved the presentation of the VIS signal accompanied by a presentation of AUD noise. AV trials involved the presentation of concordant AV signals and both AUD and VIS noises. Each run of trials consisted of 50 presentations of ‘Gary’ and 50 presentations of ‘Barry’ in each style of presentation, 300 trials in all. These were presented in random order, to prevent any practise effects from systematically impacting our data across the critical experimental conditions. Each observer completed two runs of Simultaneous trials, providing 200 responses for each style of stimulus presentation.

AUD and VIS trials could be considered as representing a conflict scenario, in that there is no coherent signal in the other sensory modality. This is unavoidable if one wants to examine the possibility of cross-modal summation, as combined modality presentations have to be compared with uni-modal presentations.

In Sequential runs of trials, two presentations were separated by an inter stimulus interval (ISI  = 3 sec). Both presentations occurred before a response was required. The three styles of presentation consisted of VIS ISI VIS, AUD ISI AUD, or combinations of AV signals. The order of presentation for the last grouping, either AUD ISI VIS or VIS ISI AUD, was randomized on a trial-by-trial basis. Other details concerning these trial runs were as per the Simultaneous runs of trials. The order in which the different types of trial run were completed was counterbalanced across observers (Simultaneous-Sequential-Simultaneous-Sequential, or Sequential-Simultaneous-Sequential-Simultaneous).

## Supporting Information

Movie S1 Example footage of actor saying “My name is Barry” with visual and audio noise
(3.72 MB MOV)

Movie S2 Example footage of actor saying “My name is Gary” with audio and visual noise
(4.12 MB MOV)

Movie S3 Example footage of actor saying “My name is Barry” with audio and visual noise
(3.97 MB MOV)

Movie S4 Example footage of actor saying “My name is Gary” with audio and visual noise.
(4.03 MB MOV)
